# Final Subcortical Motor Mapping Threshold and Overall Survival After Motor-Eloquent Glioblastoma Resection: Exploratory Analysis of Residual Fluorescence at the Motor Boundary

**DOI:** 10.3390/cancers18111741

**Published:** 2026-05-27

**Authors:** Petr Krupa, Filip Kotek, Michael Bartos, Mikulas Vachek, Marketa Krupova, Simona Paulikova, Petra Kasparova, Tomas Cesak

**Affiliations:** 1 Department of Neurosurgery, University Hospital Hradec Kralove, 500 05 Hradec Kralove, Czech Republic; filip.kotek@fnhk.cz (F.K.); michael.bartos2@fnhk.cz (M.B.); mikulas.vachek@fnhk.cz (M.V.); tomas.cesak@fnhk.cz (T.C.); 2 Department of Neurosurgery, Faculty of Medicine in Hradec Kralove, Charles University, 500 05 Hradec Kralove, Czech Republic; 3 The Fingerland Department of Pathology, Faculty of Medicine in Hradec Kralove, Charles University, University Hospital Hradec Kralove, 500 05 Hradec Kralove, Czech Republic; marketa.krupova@fnhk.cz (M.K.);; 4 Department of Oncology and Radiotherapy, University Hospital Hradec Kralove, Sokolská 581, 500 05 Hradec Kralove, Czech Republic; simona.paulikova@fnhk.cz

**Keywords:** glioblastoma, subcortical mapping, corticospinal tract, stimulation threshold, fluorescence-guided surgery, overall survival

## Abstract

**Introduction**: Subcortical motor mapping thresholds are routinely used during glioblastoma resection to reduce the risk of permanent neurological injury, but their association with survival is not well established. We evaluated whether the final subcortical motor mapping threshold recorded at the end of resection is associated with overall survival (OS) in patients undergoing mapping-guided resection of motor-eloquent glioblastoma, including exploratory evaluation by residual fluorescence adjacent to the motor pathway. **Methods**: We performed a retrospective single-center cohort study of consecutive adults with newly diagnosed IDH-wild-type glioblastoma (2018–2024) who underwent motor mapping-guided resection with a documented final subcortical stimulation threshold and received adjuvant oncological therapy. The prespecified exposure was stimulation threshold ≤ 5 mA versus >5 mA. OS was analyzed using Kaplan–Meier estimates and Cox regression. To reduce overfitting, the primary adjusted Cox model included stimulation threshold, age, and temozolomide exposure; a fully adjusted model including age, sex, extent of resection, radiotherapy regimen, and temozolomide was established for sensitivity analysis. Because proportional hazards assumptions were not fully satisfied, restricted mean survival time (RMST) differences were also estimated at prespecified horizons. **Results**: Among 36 patients, stimulation threshold ≤ 5 mA was associated with shorter OS compared with >5 mA (log-rank *p* = 0.001). In the primary adjusted Cox model, stimulation threshold > 5 mA remained associated with lower mortality risk (HR 0.35, 95% CI 0.15–0.82, *p* = 0.016); results were directionally consistent in the fully adjusted model (HR 0.24, 95% CI 0.089–0.643, *p* = 0.0046). RMST analyses favored the >5 mA group at 12, 18, and 24 months. In exploratory analyses, the association appeared most evident in patients without residual fluorescence adjacent to the motor pathway. **Conclusions**: Lower final subcortical stimulation thresholds were associated with shorter overall survival after mapping-guided resection of motor-eloquent glioblastoma. These findings suggest that the final intraoperative stimulation threshold was associated with overall survival in adjusted exploratory models and may provide prognostic information in addition to its established role in surgical safety; however, prospective validation in larger cohorts is warranted.

## Simple Summary

Glioblastoma surgery in motor-eloquent brain regions requires a careful balance between maximal tumor removal and preservation of motor function. During surgery, subcortical motor mapping is routinely used to estimate the distance between the resection margin and the corticospinal tract, but its prognostic significance remains unclear. In this retrospective single-center study of patients with newly diagnosed IDH-wild-type glioblastoma, we found that a final subcortical stimulation threshold of ≤5 mA at the end of resection was associated with significantly shorter overall survival compared with higher thresholds. This association remained present after adjustment for major clinical and treatment factors and appeared most evident in patients without residual fluorescence at the motor boundary. These findings suggest that the final stimulation threshold may carry clinically relevant prognostic information in addition to its established role in surgical safety, although prospective validation is needed.

## 1. Introduction

Glioblastoma (GBM) remains the most aggressive primary malignant brain tumor in adults [1]. GBM is characterized by diffuse infiltration, marked intratumoral heterogeneity, treatment resistance, and near-universal recurrence, all of which contribute to its highly aggressive clinical course [2,3]. Despite modern multidisciplinary care, outcomes are still dominated by early progression and treatment resistance [4,5]. Current standard therapy for most patients includes maximal safe resection followed by radiotherapy with concomitant and adjuvant temozolomide (TMZ), a regimen that established a survival benefit over radiotherapy alone and continues to anchor contemporary practice [6].

Extent of resection is one of the few modifiable determinants of outcome in GBM, yet the oncologic goal of cytoreduction is frequently constrained by proximity to eloquent pathways [7]. This onco-functional dilemma is particularly relevant in motor-eloquent glioma surgery, where preservation of neurological function, functional independence, and timely postoperative therapy must be balanced against the survival benefit of maximal cytoreduction [8,9,10]. Recent work has also emphasized the importance of individualized functional mapping and tract-oriented surgical planning in defining the true resection boundary in eloquent tumors [11,12]. Intraoperative neurophysiological mapping and monitoring have therefore become integral to surgery near the corticospinal tract (CST) [13,14].

Subcortical motor mapping with monopolar stimulation provides a quantitative estimate of functional proximity to the CST. Multiple studies support a near-linear relationship between stimulation intensity and tract distance, commonly approximated as ~1 mA per ~1 mm, enabling threshold-guided decisions at the functional boundary of resection [15,16]. Although thresholds are routinely used as safety criteria, their potential prognostic implications have received comparatively limited emphasis [17]. Recent evidence in motor-pathway-involving gliomas suggests that lower minimal subcortical thresholds predict not only postoperative motor deficits but also shorter survival, raising the possibility that intraoperative thresholds may encode the degree of onco-functional constraint encountered during resection [17].

In parallel, 5-aminolevulinic acid (5-ALA) fluorescence-guided surgery increases the likelihood of gross-total resection and has become widely used in GBM [18,19]. Notably, residual intraoperative fluorescence has been associated with worse overall survival even after radiographic complete resection of contrast-enhancing tumor, indicating that biologically relevant tumor may remain at the surgical margin [20,21]. This observation is particularly relevant at motor boundaries, where resection may terminate at the intersection of oncologic target and functional risk. However, key uncertainties remain for GBM: whether a clinically interpretable threshold cutoff can stratify overall survival in a homogeneous GBM cohort; whether the association persists after accounting for major prognostic and treatment variables; and how the signal behaves in subsets defined by the radicality of resection and fluorescence at the motor boundary.

Against this background, the prognostic significance of intraoperative stimulation thresholds remains incompletely defined, particularly when thresholds are interpreted in relation to the final functional margin at which resection is terminated. Unlike prior studies focused on minimal thresholds recorded at any point during surgery, the present study evaluates the final subcortical stimulation threshold at the functional stopping boundary. We selected a threshold of 5 mA a priori as a clinically interpretable cutoff, because thresholds in this range are commonly regarded intraoperatively as indicating close corticospinal tract proximity and frequently influence decisions on whether further resection may carry unacceptable motor risk. Our objective was therefore not to derive an optimal cut point from the present dataset but to examine whether a familiar decision-relevant threshold was associated with overall survival in a homogeneous glioblastoma cohort. We hypothesized that the final stimulation threshold would be associated with the degree of functional constraint encountered during maximal safe tumor removal.

## 2. Methods

### 2.1. Study Design, Setting, and Ethics

This was a single-center retrospective cohort of consecutive adults with histologically confirmed GBM treated at the Department of Neurosurgery, University Hospital Hradec Kralove, Czech Republic, between 1 January 2018 and 31 December 2024. Personal data and surgical reports were abstracted from electronic records. The study was approved by the Local Ethics Committee (202602 P08) and conducted in accordance with the Declaration of Helsinki [22].

Patients were eligible if they were ≥18 years old, had newly diagnosed IDH-wild-type GBM according to WHO 2021 criteria, and underwent initial monitored resection with a documented final subcortical motor mapping threshold followed by postoperative adjuvant oncological therapy. Patients were excluded if they did not receive postoperative adjuvant oncological therapy (n = 1) due to poor postoperative performance status. Standard management consisted of surgery with the goal of maximal safe resection, followed by radiotherapy. Postoperative radiotherapy was categorized as 60 Gy in 30 fractions or 45 Gy in 15 fractions with concomitant temozolomide (TMZ; 75 mg/m^2^ daily) and subsequent adjuvant TMZ 150–200 mg/m^2^ on days 1–5 every 28 days, in accordance with our local Stupp-based protocol. The radiotherapy regimen was recorded from the oncology treatment summary and coded as a binary variable for analysis. Follow-up imaging was performed per routine practice at 3-month intervals during scheduled oncology visits. Reporting followed the Strengthening the Reporting of Observational Studies in Epidemiology (STROBE) guideline for cohort studies [23].

### 2.2. Intraoperative Motor Mapping and Stimulation Protocol

Total intravenous anesthesia was used, and neuromuscular blockade was minimized after intubation to maintain stable MEP/EMG monitoring conditions. Intraoperative motor pathway monitoring and mapping were performed using an inomed neuromonitoring system (inomed Medizintechnik GmbH, Emmendingen, Germany).

For cortical stimulation, a 2-point strip electrode was used. A 4-point strip electrode was placed for continuous electrocorticography (cEEG) monitoring. Subcortical mapping was performed at the resection cavity margin using a unipolar suction stimulation electrode with constant-current stimulation, typically referenced to a remote electrode (e.g., Fz). A stimulation site was considered positive when it produced a reproducible, time-locked motor response recorded on EMG and/or MEP channels in predefined contralateral upper- and/or lower-extremity muscles. Target muscle sets were selected according to tumor laterality and anticipated CST risk.

Stimulation was delivered with the inomed HC Stimulator using a short-train paradigm consisting of 6 pulses with negative polarity, pulse width 500 µs, and interstimulus interval 4.0 ms (intratrain frequency 250 Hz). The train repetition rate was 1.5 Hz. Stimulation intensity was titrated in milliamperes to identify the lowest current producing a reproducible motor response on EMG/MEP monitoring. The stimulator was configured with a maximum output of 20 mA and a maximum compliance voltage of 200 V.

Subcortical motor mapping was performed continuously during resection using a monopolar suction stimulation electrode directed toward the corticospinal tract along the full extent of the resection wall, according to the evolving surgical anatomy and operative workflow. As a result, multiple subcortical sites were functionally assessed throughout the procedure. For the purposes of this study, the analyzed exposure was defined as the lowest current eliciting a reproducible motor response at the end of resection, at the site judged intraoperatively to represent the functional stopping margin closest to the corticospinal tract. The value used for analysis was abstracted from the explicit note in the operative report. Thresholds documented as exceeding the maximal tested intensity were recorded as “>15 mA” and were top-coded to 16 mA to allow inclusion as numeric while preserving ordering. Stimulation thresholds were interpreted as an intraoperative surrogate of functional proximity to the corticospinal tract (commonly approximated as ~1 mA per ~1 mm, acknowledging technique-dependent variability).

### 2.3. Clinical Covariates and Endpoints

Clinical variables included sex, age at operation, pre-operative tumor volume, and extent of resection (EOR). EOR was classified as complete resection (CR), near-total (NTR), subtotal (STR), or partial resection (PR) on early post-op contrast-enhanced MRI performed within 72 h after surgery according to Karschnia [24]. Residual fluorescence adjacent to the motor pathway was recorded as an intraoperative variable referring to visible 5-ALA fluorescence persisting at the functionally constrained motor boundary at the end of resection. Given the retrospective design and the subjective nature of fluorescence intensity grading, fluorescence was analyzed as a binary variable (present/absent) only. Motor deficits were graded on an ordinal 0–4 scale at two time points (immediate postoperative and discharge): 0 = no paresis, 1 = mild, 2 = moderate, 3 = severe, 4 = plegia. Moreover, Karnofsky Performance Status (KPS) and comorbidities were also noted. Given the modest sample size, KPS was treated as a descriptive clinical characteristic and was not incorporated into the primary multivariable survival models in order to reduce overfitting. The primary endpoint was overall survival (OS) in days, defined from operation date to death; patients alive at database lock were administratively censored on 5 January 2026 during the last scheduled follow-up (n = 5). Given the limited sample size, the primary adjusted Cox model included stimulation threshold, age, and temozolomide exposure to reduce overfitting. A fully adjusted model including age, sex, extent of resection, radiotherapy regimen, and temozolomide exposure was performed as a sensitivity analysis.

### 2.4. Statistical Analysis

Overall survival (OS) was defined as the time from surgery to death from any cause or censoring at last follow-up (5 January 2026). Survival distributions were estimated using the Kaplan–Meier method and summarized as median OS with 95% confidence intervals (CIs). Group comparisons were performed using log-rank tests.

The primary exposure was the final subcortical motor stimulation threshold, analyzed using a prespecified cutoff of ≤5 mA versus >5 mA. This threshold was selected a priori for clinical interpretability, as values around 5 mA are widely used in subcortical motor mapping as a practical marker of close corticospinal tract proximity and may influence the decision to terminate further resection. The cutoff was not derived from outcome optimization within the present dataset. Hazard ratios (HRs) with 95% CIs were estimated using Cox proportional hazards regression. Given the limited sample size, the primary adjusted Cox model was prespecified as a parsimonious model including stimulation threshold, age at surgery, and temozolomide exposure (any concurrent and/or adjuvant treatment). A fully adjusted model including age, sex, extent of resection (percent of contrast-enhancing tumor removed), radiotherapy regimen (60 Gy/30 fractions vs. 45 Gy/15 fractions), and temozolomide exposure was performed as a sensitivity analysis. Temozolomide exposure was defined as actually receiving concurrent and/or adjuvant TMZ, as documented in the oncology treatment records, rather than intended treatment.

Because proportional hazards assumptions were not fully satisfied for the stimulation-threshold effect, restricted mean survival time (RMST) differences between groups were estimated as a complementary, assumption-robust measure. RMST differences were evaluated at prespecified truncation times of 12, 18, and 24 months (365, 548, and 730 days), with 95% CIs obtained by bootstrap resampling.

To account for potential differences in baseline hazard by radiotherapy schedule in exploratory subgroup analyses, additional Cox models stratified by radiotherapy regimen were fitted where specified. Subgroup analyses stratified by residual fluorescence adjacent to the motor pathway and postoperative motor deficit were considered exploratory and interpreted accordingly.

Continuous variables were compared between groups using the Mann–Whitney U test, and categorical variables were compared using Fisher’s exact test. All tests were two-sided with a significance threshold of α = 0.05. Analyses were performed using a reproducible script-based workflow in Python (version 3.12.), and Cox models used the Breslow method for handling ties.

## 3. Results

### 3.1. Study Cohort and Follow-Up

**Figure 1:**
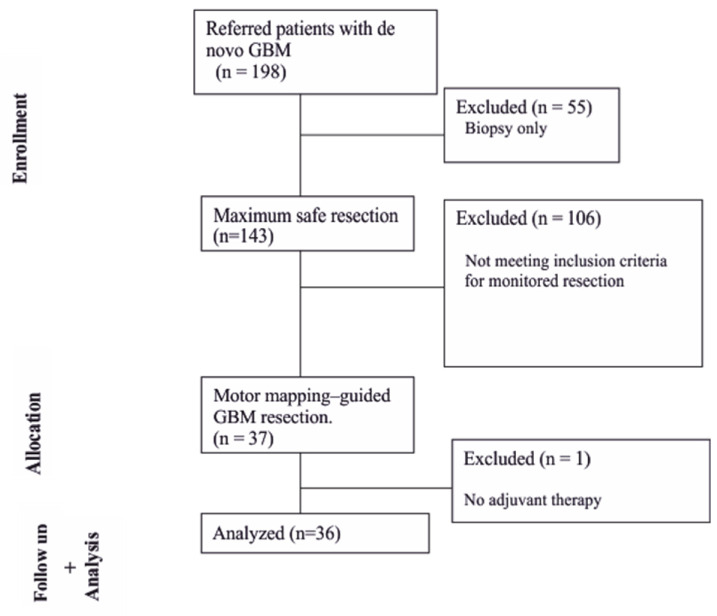
STROBE flow diagram of cohort selection.

Of 143 surgically treated GBM patients, 37 underwent monitored motor-eloquent resection with a documented final subcortical motor mapping threshold. One patient did not receive postoperative adjuvant therapy and was excluded, leaving 36 patients for the prespecified analytical cohort (Figure 1). At the database lock, 31 deaths had occurred, and 5 patients were administratively censored.

### 3.2. Patient Characteristics

In the study cohort (n = 36), patients were evenly distributed between stimulation threshold groups (≤5 mA: n = 18; >5 mA: n = 18). Median age was similar (≤5 mA: 68 years; >5 mA: 66 years; *p* = 0.635), and sex distribution was identical (44.4% male in both groups). Median extent of resection of the contrast-enhancing tumor was high and comparable (median 99.7% in both groups; *p* = 0.946). Adjuvant treatment variables were numerically imbalanced, with the >5 mA group more often receiving the 60 Gy/30 fraction regimen (14/18, 77.8% vs. 9/18, 50.0%) and any temozolomide exposure (17/18, 94.4% vs. 12/18, 66.7%) than the ≤5 mA group (Table 1). Because these treatment differences could confound survival analyses, radiotherapy regimen and temozolomide exposure were incorporated into adjusted models. Patients in the ≤5 mA group had higher postoperative motor deficit grades (median 2 vs. 0, *p* < 0.001), with substantial improvement by discharge. Residual fluorescence adjacent to the motor pathway was present in 10/36 (27.8%) patients overall (≤5 mA: 38.9% vs. >5 mA: 16.7%; *p* = 0.264). The resection radicality group distribution did not differ between stimulation groups.

### 3.3. Overall Survival by Stimulation Threshold

**Figure 2:**
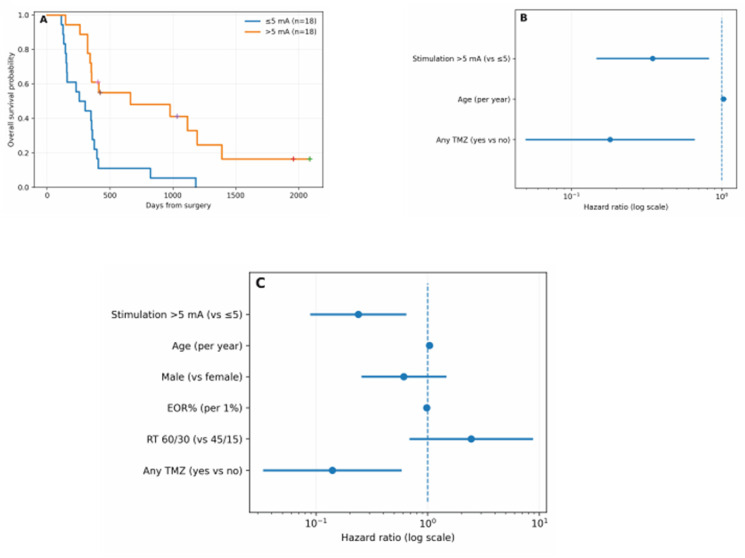
Final stimulation threshold and overall survival: (**A**) Kaplan–Meier overall survival (OS) curves comparing patients with final stimulation threshold ≤ 5 mA versus >5 mA. Tick marks indicate censored observations. OS differed significantly between groups (log-rank *p* = 0.0010). Median OS was 255 days (95% CI 152–359) for ≤5 mA and 663 days (95% CI 342–1194) for >5 mA. (**B**) Parsimonious Cox proportional hazards model including final stimulation threshold (>5 vs. ≤5 mA), age, and temozolomide (TMZ) exposure. Final stimulation threshold > 5 mA remained associated with improved OS after adjustment for age and temozolomide exposure (HR 0.35, 95% CI 0.15–0.82, *p* = 0.016). (**C**) Fully adjusted Cox model including final stimulation threshold, age, sex, extent of resection (EOR, %), radiotherapy regimen, and TMZ exposure. Final stimulation threshold > 5 mA remained associated with improved OS (HR 0.24, 95% CI 0.09–0.64, *p* = 0.0046).

Kaplan–Meier analysis demonstrated significantly worse overall survival (OS) in patients with a final stimulation threshold ≤ 5 mA compared with >5 mA (log-rank *p* = 0.001). Median OS was 255 days (95% CI 152–359) in the ≤5 mA group versus 663 days (95% CI 342–1194) in the >5 mA group (Figure 2A).

In univariable Cox regression, a stimulation threshold > 5 mA was associated with significantly lower mortality risk (HR 0.29, 95% CI 0.14–0.64, *p* = 0.0019).

In a prespecified parsimonious multivariable Cox model adjusting for age and TMZ exposure, stimulation threshold remained associated with overall survival, with thresholds > 5 mA associated with lower mortality risk (HR 0.35 (95% CI 0.15–0.82), *p* = 0.016). TMZ exposure was also independently associated with improved survival (HR: 0.18 (95% CI 0.049–0.663), *p* = 0.0099), while age was not statistically significant (Figure 2B).

In a fully adjusted multivariable model including age, sex, EOR%, RT regimen, and TMZ exposure, the association between stimulation threshold and OS remained statistically significant (HR 0.24 (95% CI 0.089–0.643), *p* = 0.0046). TMZ exposure also remained independently associated with improved survival (HR: 0.14 (95% CI 0.034–0.584), *p* = 0.0070), while age, sex, EOR%, and RT regimen were not statistically significant in this cohort (Table 2, Figure 2C). For details, see Supplementary Table S1.

### 3.4. Sensitivity Analysis Addressing Non-Proportional Hazards

**Figure 3:**
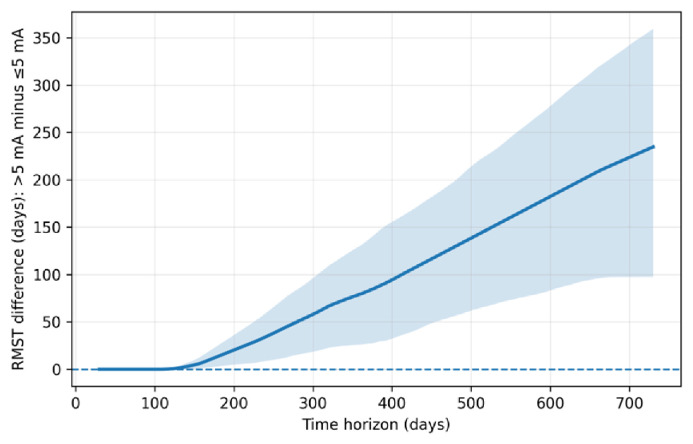
Restricted mean survival time analysis. Restricted mean survival time (RMST) difference for OS comparing >5 mA versus ≤5 mA stimulation thresholds across time horizons (positive values favor > 5 mA). The solid line represents the estimated RMST difference, the shaded area indicates the bootstrap 95% confidence interval, and the horizontal dashed line indicates no difference between groups. Estimated RMST benefit for >5 mA was +81 days at 12 months, +160 days at 18 months, and +235 days at 24 months.

Because stimulation threshold effects may vary over time, restricted mean survival time (RMST) differences were evaluated at prespecified horizons. RMST favored the >5 mA group at all horizons: +81 days at 12 months (bootstrap 95% CI 28–134), +160 days at 18 months (95% CI 70–245), and +235 days at 24 months (95% CI 98–359), supporting a clinically meaningful survival advantage associated with >5 mA independent of proportional hazards assumptions (Figure 3).

### 3.5. Impact of Fluorescence Adjacent to the Motor Pathway Status: Post Hoc Subgroup Analyses

**Figure 4:**
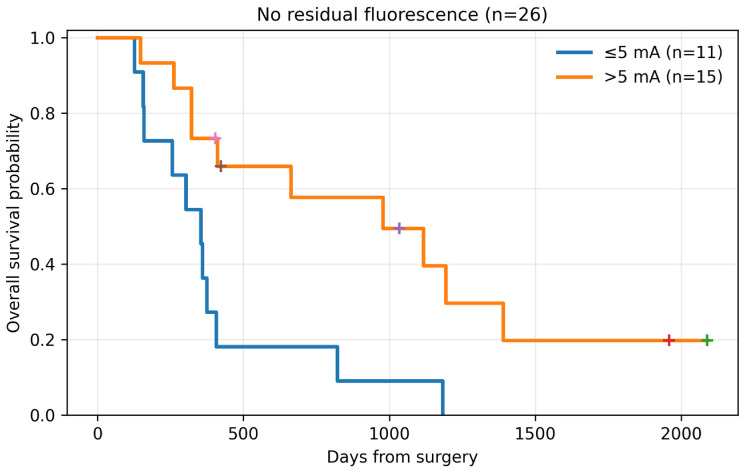
Overall survival by stimulation threshold in patients without residual fluorescence. Kaplan–Meier OS curves stratified by final stimulation threshold (≤5 mA vs. >5 mA) among patients without residual fluorescence adjacent to the motor pathway (n = 26). Tick marks indicate censored observations. OS differed significantly between groups (log-rank *p* = 0.0049). Median OS was 354 days for ≤5 mA and 978 days for >5 mA.

In the subgroup without residual fluorescence adjacent to the motor pathway (n = 26), OS separation by stimulation threshold remained pronounced (median OS 354 vs. 978 days for ≤5 vs. >5 mA; log-rank *p* = 0.0049; univariable Cox HR 0.28, 95% CI 0.11–0.72, *p* = 0.008) (Figure 4).

In the subgroup with residual fluorescence adjacent to the motor pathway (n = 10), OS was numerically longer in the >5 mA group than in the ≤5 mA group (median OS 351 vs. 152 days), but this exploratory comparison did not reach statistical significance (univariable Cox HR 0.55, 95% CI 0.13–2.29, *p* = 0.415), consistent with the very limited statistical power of this subgroup. In this fluorescence-positive subgroup, MRI-based EOR was classified as CR in 2 patients, NTR in 5, STR in 2, and PR in 1, underscoring that residual fluorescence at the motor boundary and postoperative radiographic EOR were not equivalent variables.

### 3.6. Postoperative Motor Deficit and Overall Survival

**Figure 5:**
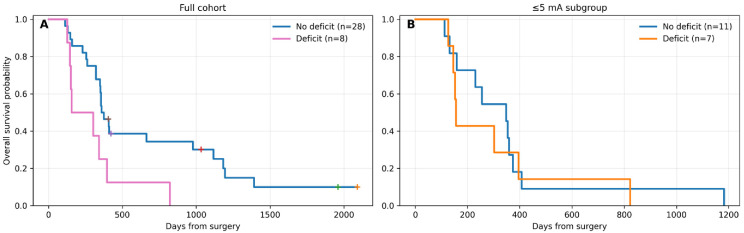
Discharge motor deficit and overall survival: (**A**) Kaplan–Meier OS curves comparing patients with versus without a discharge motor deficit in the full cohort (any deficit, defined as discharge motor grade > 0, vs. none, grade 0). Tick marks indicate censored observations. Presence of a discharge motor deficit was associated with worse OS (log-rank *p* = 0.0147; HR 2.73, 95% CI 1.18–6.36). (**B**) Kaplan–Meier OS curves comparing discharge motor deficit groups within the ≤5 mA stimulation subgroup. No significant difference in OS was observed (log-rank *p* = 0.607; HR 1.29, 95% CI 0.49–3.43). Interaction testing did not support effect modification of the stimulation threshold–survival association by discharge deficit (*p* for interaction = 0.350).

Motor deficit at discharge was associated with worse overall survival in the full cohort (log-rank *p* = 0.015; HR 2.73, 95% CI 1.18–6.36) (Figure 5A). However, within the ≤5 mA subgroup, discharge deficit was not associated with survival (log-rank *p* = 0.607; HR 1.29, 95% CI 0.49–3.43) (Figure 5B). Furthermore, interaction testing provided no evidence that discharge deficit modified the relationship between stimulation threshold and survival (interaction *p* = 0.350). These findings indicate that the association between stimulation threshold and survival was not fully accounted for by discharge motor deficit in this cohort.

## 4. Discussion

In this single-center retrospective cohort of motor-eloquent GBM resections performed with intraoperative motor pathway monitoring/mapping, a low final stimulation threshold (≤5 mA) was associated with shorter overall survival compared with thresholds > 5 mA. The association remained directionally consistent across complementary analyses, including adjusted Cox models and restricted mean survival time (RMST) estimates intended to reduce reliance on proportional hazards assumptions. In addition, the association persisted in the subgroup without residual 5-ALA fluorescence adjacent to the motor pathway. Collectively, these findings suggest that the final stimulation threshold—primarily used intraoperatively for safety—may also reflect the degree of functional constraint encountered during resection of motor-eloquent GBM.

Survival in GBM is strongly influenced by tumor biology and the ability to deliver effective adjuvant therapy. The current therapeutic backbone—maximal safe resection followed by radiotherapy with concomitant and adjuvant temozolomide—was established in the landmark randomized trial by Stupp and colleagues [6] and remains the main comparator for outcome interpretation in contemporary series. Within this framework, intraoperative factors that shape the achievable extent of resection and/or postoperative recovery may have downstream prognostic relevance.

Subcortical motor mapping thresholds are widely used as a practical surrogate for functional proximity to the corticospinal tract (CST) [15,25,26]. Across commonly applied monopolar short-train paradigms, an approximately linear relationship between stimulation intensity and CST distance is often used heuristically (e.g., ~1 mA ≈ ~1 mm), while recognizing variability related to stimulation parameters, probe geometry, anesthetic conditions, and tissue conductivity [25]. Continuous/dynamic mapping approaches further reinforce the clinical role of this metric by integrating stimulation into the resection workflow itself (e.g., suction-probe mapping), allowing iterative “functional boundary” assessment as the cavity deepens. From a surgical standpoint, the final threshold recorded at the end of resection can be interpreted as a quantitative marker of how narrowly the operation was limited by CST proximity at the point where resection stopped [27].

Most prior work has emphasized thresholds as predictors of postoperative motor morbidity, whereas survival has less often been examined as a primary endpoint [28]. In our cohort, discharge motor deficit was associated with overall survival in the full population; however, the association between stimulation threshold and survival was not fully accounted for by discharge motor deficit in this dataset. Specifically, within the ≤5 mA subgroup, discharge motor deficit was not associated with survival, and interaction testing did not support effect modification by discharge deficit. This argues against a simple interpretation whereby shorter survival in the low-threshold group is driven mainly by clinically apparent postoperative weakness alone.

A plausible interpretation of the observed association is that a low final stimulation threshold reflects termination of resection at a very narrow functional margin, leaving limited opportunity for further cytoreduction without unacceptable motor risk [15]. In this sense, the final threshold may function less as a direct biological marker than as an intraoperative indicator of onco-functional constraint. Conversely, higher final thresholds may reflect a greater functional distance from the corticospinal tract at the stopping boundary and therefore a wider margin for maximal safe tumor removal. This interpretation is consistent with the broader concept that the oncological value of resection depends not only on cytoreduction itself but also on the preservation of neurological function and the ability to receive adjuvant therapy. In addition, glioma cells are known to migrate along white-matter structures, providing a biologically plausible framework for the possibility that tumors closely related to major motor tracts may also differ in infiltrative behavior [29,30,31]. However, these mechanisms cannot be established from the present retrospective dataset and should be regarded as plausible interpretations rather than direct evidence.

Recent data support the possibility that stimulation thresholds encode information beyond immediate surgical risk. Ren and colleagues reported that lower minimal subcortical thresholds were associated with adverse neurological and oncologic endpoints in gliomas involving motor pathways and proposed a clinically relevant low-threshold range [17]. Our findings are consistent with this direction of association and extend the concept by focusing on a pragmatic metric—the final threshold at resection completion—using a prespecified cutoff (≤5 mA) that is familiar in CST-adjacent surgery.

In exploratory subgroup analyses, the relationship between threshold and survival appeared most evident in patients without residual 5-ALA fluorescence adjacent to the motor pathway. This pattern is clinically plausible given evidence that residual fluorescence at the margin can itself be associated with inferior survival, consistent with biologically meaningful residual tumor [18,20]. When fluorescence is absent at the motor boundary, stimulation threshold may more cleanly reflect the functional limit of resection; when fluorescence persists, the prognostic contribution of residual tumor at the interface may be larger and could reduce the ability to detect additional effects of threshold, particularly in small subgroups [32].

Muscas et al. reported 5-ALA–guided resections for GBM involving motor pathways using stimulation-based monitoring and MEPs, with resection commonly interrupted when direct stimulation at 5 mA was positive or when MEP amplitude declined [33]. Their approach supports the clinical relevance of a 5 mA boundary as a practical CST proximity zone. In this context, our data suggest that the final threshold at which resection is ultimately terminated may have prognostic correlates, although the mechanism cannot be determined from this study.

This study has several limitations. The retrospective single-center design limits control over confounding and the completeness of covariate capture. The final stimulation threshold was recorded as part of routine clinical documentation rather than within a prospective protocol specifically designed for prognostic analysis. The cohort size was modest and derived from a single center, limiting precision and subgroup inference. A central limitation is the absence of sufficiently complete MGMT promoter methylation data for robust inclusion in the present analyses. This is particularly relevant because MGMT status influences temozolomide responsiveness and overall survival in glioblastoma. In addition, treatment imbalances between groups remained present despite adjustment. Although these variables were incorporated into adjusted models, statistical adjustment cannot fully eliminate the possibility that treatment imbalance contributed to the observed survival differences in a retrospective cohort of this size. Moreover, because temozolomide exposure was defined according to actually received treatment, this variable may also partly reflect postoperative status, early death, or treatment eligibility, introducing potential post-baseline treatment bias. Finally, stimulation thresholds are affected by technical factors (probe contact, stimulation paradigm, and anesthetic conditions), and external validation will require protocol standardization. Prospective multi-institutional studies with molecular characterization and standardized mapping protocols will be needed to confirm generalizability and clarify interpretation.

Clinically, the final mapping threshold is available intraoperatively and may contribute to postoperative risk stratification and counseling when interpreted alongside established prognostic factors. If validated, threshold-based stratification could support risk adjustment in comparative studies of surgical strategies for motor-eloquent GBM and may help frame postoperative management discussions in selected patients.

## 5. Conclusions

This study extends the existing literature by evaluating the final subcortical stimulation threshold recorded at the point where resection stops, reflecting the functional boundary that ultimately constrained tumor removal. Using a prespecified and clinically interpretable cutoff (≤5 mA), we observed that lower final thresholds were associated with shorter overall survival in adjusted exploratory analyses of major clinical and treatment variables. A notable innovative aspect of this study is the focus on the final subcortical stimulation threshold at resection completion, rather than the minimum threshold observed at any point during surgery, as a pragmatic intraoperative summary of the functional boundary that ultimately constrained tumor removal. In exploratory subgroup analyses, the association appeared most evident in patients without residual fluorescence adjacent to the motor pathway, suggesting that stimulation threshold may provide complementary prognostic information when fluorescence-defined residual tumor at the functional margin is absent.

These findings support the hypothesis that intraoperative neurophysiologic metrics may have prognostic correlates beyond their established safety role. However, given the retrospective design and modest cohort size, these observations should be considered hypothesis-generating and require validation in larger, prospectively characterized cohorts with integrated molecular data.

## Figures and Tables

**Table 1:** Baseline characteristics. Extent of resection categories: CR (complete resection, 100%), NTR (≥95%), STR (≥80%), PR (1–79%).

Characteristic	≤5 mA (n = 18)	>5 mA (n = 18)
Patients	18	18
Female, n (%)	10 (55.6)	10 (55.6)
Male, n (%)	8 (44.4)	8 (44.4)
Age, median (IQR), y	67.5 (59.2–74.0)	66.0 (61.2–71.2)
Pre-op tumor volume, median (IQR), cm^3^	16.5 (6.5–31.1)	15.0 (9.0–34.2)
Extent of resection—CR, n (%)	9 (50.0)	9 (50.0)
Extent of resection—NTR, n (%)	7 (38.9)	5 (27.8)
Extent of resection—STR, n (%)	2 (11.1)	3 (16.7)
Extent of resection—PR, n (%)	0 (0.0)	1 (5.6)
Deaths (events), n	18	13
Censored, n	0	5
K-M median OS, days	255	663
Any TMZ exposure—yes, n (%)	12 (66.7)	17 (94.4)
Concomitant TMZ—yes, n (%)	8 (44.4)	16 (88.9)
Radiotherapy 60 Gy/30 fr, n (%)	9 (50.0)	14 (77.8)
Preoperative KPS, median	70	70
Postoperative KPS, median	65	70

**Table 2:** Cox proportional hazards regression for overall survival.

Variable	Univariable HR (95% CI)	*p*	Parsimonious Model HR (95% CI)	*p*	Fully Adjusted Model HR (95% CI)	*p*
Stimulation threshold > 5 mA (vs. ≤5 mA)	0.29 (0.14–0.64)	0.0019	0.35 (0.15–0.82)	0.016	0.24 (0.09–0.64)	0.0046
Age (per year)	—	—	1.03 (0.98–1.08)	0.216	1.04 (0.99–1.09)	0.133
Temozolomide exposure (any vs. none)	—	—	0.18 (0.05–0.66)	0.0099	0.14 (0.03–0.58)	0.0070
Sex (female vs. male)	—	—	—	—	1.64 (0.68–3.93)	0.270
Extent of resection (per 1%)	—	—	—	—	0.98 (0.94–1.02)	0.299
Radiotherapy regimen (60/30 vs. 45/15)	—	—	—	—	2.45 (0.69–8.78)	0.167

## Data Availability

De-identified data are available upon reasonable request.

## Supplementary Materials

The following supporting information can be downloaded at: https://www.mdpi.com/article/10.3390/cancers18111741/s1, Supplementary Table S1: Cox proportional hazards regression models for overall survival. Full regression output for parsimonious and fully adjusted Cox models, including beta coefficients, standard errors, hazard ratios, 95% confidence intervals, and *p*-values. The parsimonious model was adjusted for age and temozolomide exposure. The fully adjusted model additionally included sex, extent of resection, and radiotherapy regimen.
